# Differential Effects of Electronic Hookah Vaping and Traditional Combustible Hookah Smoking on Oxidation, Inflammation, and Arterial Stiffness

**DOI:** 10.1016/j.chest.2021.07.027

**Published:** 2021-07-21

**Authors:** Mary Rezk-Hanna, Rajat Gupta, Charlie O. Nettle, Daniel Dobrin, Chiao-Wei Cheng, Angelica Means, Mary-Lynn Brecht, Donald P. Tashkin, Jesus A. Araujo

**Affiliations:** a School of Nursing, University of California, Los Angeles, CA; b Division of Cardiology Department of Medicine, David Geffen School of Medicine, University of California, Los Angeles, CA; c Division of Pulmonary and Critical Care Medicine, Department of Medicine, David Geffen School of Medicine, University of California, Los Angeles, CA; d Molecular Biology Institute, University of California, Los Angeles, CA; e Department of Environmental Health Sciences, Fielding School of Public Health, University of California, Los Angeles, CA

**Keywords:** arterial stiffness, electronic hookah, electronic water pipe, hookah smoking, inflammation, oxidation, CO, carbon monoxide, CV, coefficient of variation, e-hookah, electronic hookah, ENDS, electronic nicotine delivery system, HDL, high-density lipoprotein, hsCRP, high sensitivity C-reactive protein, PON-1, paraoxonase-1, PWV, pulse wave velocity, TNFα, tumor necrosis factor α

## Abstract

**Background:**

Traditional hookah smoking has grown quickly to become a global tobacco epidemic. More recently, electronic hookahs (e-hookahs)—vaped through traditional water pipes—were introduced as healthier alternatives to combustible hookah. With combustible tobacco smoking, oxidative stress, inflammation, and vascular stiffness are key components in the development and progression of atherosclerosis. The comparable effects of hookah are unknown.

**Research Question:**

What is the differential acute effect of e-hookah vaping vs combustible hookah smoking on oxidation, inflammation, and arterial stiffness?

**Study Design and Methods:**

In a randomized crossover design study, among a cohort of 17 healthy young adult chronic hookah smokers, we investigated the effect of e-hookah vaping and hookah smoking on measures of conduit arterial stiffness, including carotid-femoral pulse wave velocity (PWV), augmentation index-corrected for heart rate before and after a 30-min exposure session. We assessed a panel of circulating biomarkers indicative of inflammation and oxidants and measured plasma nicotine and exhaled carbon monoxide (CO) levels before and after the sessions.

**Results:**

e-Hookah vaping tended to lead to a larger acute increase in PWV than hookah smoking (mean ± SE: e-hookah, +0.74 ± 0.12 m/s; combustible hookah, +0.57 ± 0.14 m/s [*P* < .05 for both]), indicative of large artery stiffening. Compared with baseline, only e-hookah vaping induced an acute increase in augmentation index (e-hookah, +5.58 ± 1.54% [*P* = .004]; combustible hookah, +2.87 ± 2.12% [*P* = not significant]). These vascular changes were accompanied by elevation of the proinflammatory biomarkers high-sensitivity C-reactive protein, fibrinogen, and tumor necrosis factor α after vaping (all *P* < .05). No changes in biomarkers of inflammation and oxidants were observed after smoking. Compared with baseline, exhaled CO levels were higher after smoking than after vaping (+36.81 ± 6.70 parts per million vs –0.38 ± 0.22 parts per million; *P* < .001), whereas plasma nicotine concentrations were comparable (+6.14 ± 1.03 ng/mL vs +5.24 ± 0.96 ng/mL; *P* = .478).

**Interpretation:**

Although advertised to be “safe,” flavored e-hookah vaping exerts injurious effects on the vasculature that are, at least in part, mediated by inflammation.

**Trial Registry:**

ClinicalTrials.gov; No.: NCT03690427; URL: www.clinicaltrials.gov

## Graphical Abstract

**Figure:**
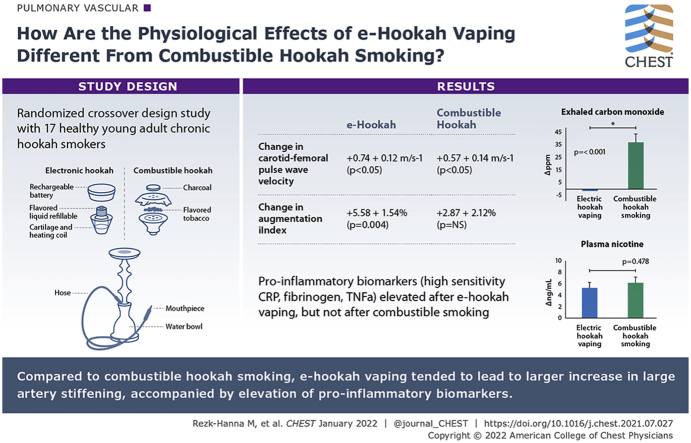

FOR EDITORIAL COMMENT, SEE PAGE 13

Hookah (ie, water-pipe) smoking has grown quickly to become a major global tobacco epidemic.^1^ Contributing to this popularity is the belief that traditional charcoal-heated hookah smoke is detoxified as it passes through the water-filled base, rendering hookah smoking a safer tobacco alternative.^2^ In 2014, electronic hookahs (e-hookahs) were introduced as healthier alternatives to hookah smoking.^3^^,^^4^ Data from wave 1 of the Population Assessment of Tobacco and Health Study (2013-2014) show that among adults 18 to 24 years of age, 18.2% reported current hookah smoking.^5^ Wave 2 data from the Population Assessment of Tobacco and Health Study (2014-2015) show that 7.7% of youth reported ever e-hookah use.^6^ Among adults, 4.6% reported ever e-hookah use, and of these, more than one-quarter (26.8%) reported current use.^6^

With traditional hookah, in addition to tobacco combustion products, smokers are exposed to charcoal combustion products from the burning charcoal used to heat the fruit-flavored tobacco product (Fig 1A). These charcoal combustion products include: (1) concentrated carbon-rich nanoparticles, which have a mean aerodynamic diameter that is an order of magnitude smaller than the nanoparticles in cigarette smoke and are postulated to be more potent oxidants to the vasculature^7^^,^^8^; and (2) large amounts of carbon monoxide (CO), a putative vasodilator molecule. e-Hookahs are a new category of vaping devices in which e-bowls are combined with and placed on traditional water pipes, allowing the aerosol to pass through a water-filled base before being inhaled (Fig 1B). Because of the absence of combustion, e-hookahs have been marketed as a “safe” alternative to hookah smoking. However, because of the use of the heating element, e-hookahs deliver flavored nicotine by creating an aerosol of nanoparticles and other free radicals that may increase cardiovascular disease risk by activating inflammation and oxidative stress and impairing aortic elastic properties, leading to arterial stiffness. Indeed, substantial evidence suggests that inflammation and oxidative stress are central to the ability of cigarette smoking to cause atherosclerotic vascular disease.^9^

**Figure 1:**
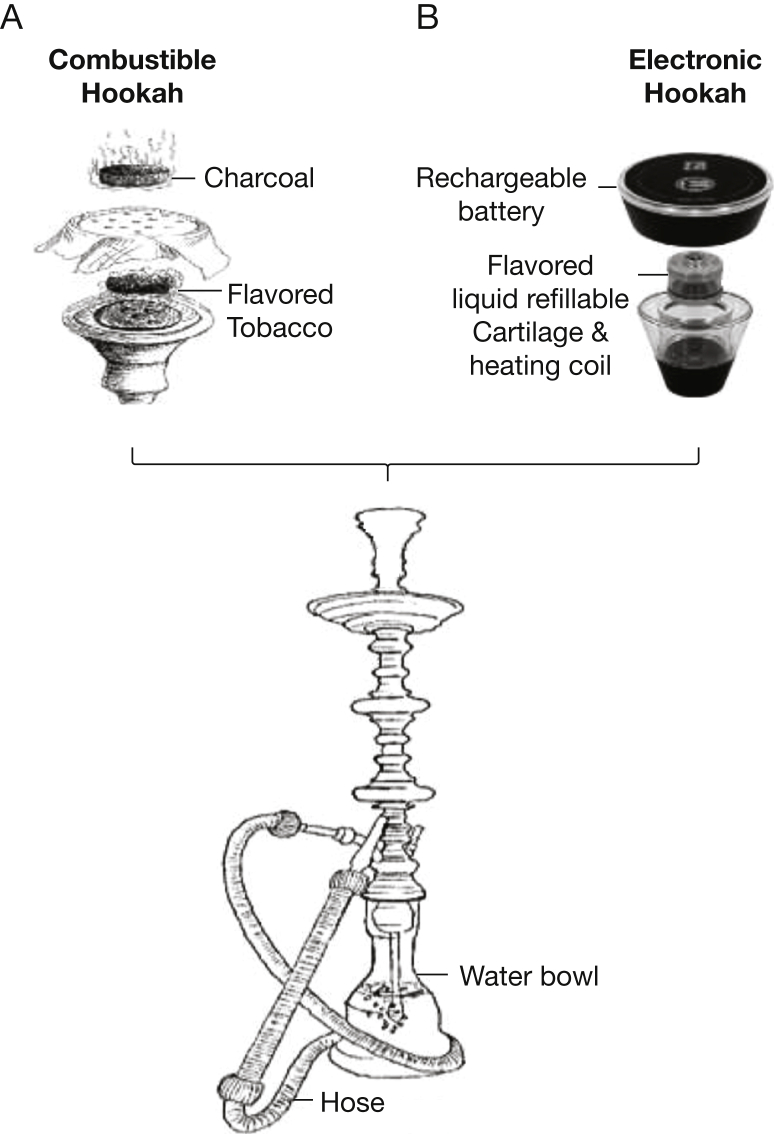
A, B, Diagram showing combustible hookah vs e-hookah components. A, With combustible hookah, flavored tobacco is placed inside the bowl and heated with charcoal. As the smoker inhales through the hose, the negative pressure generated heats up the charcoal, which chars the flavored tobacco and causes the smoke to pass through the water and into the user’s mouth. B, With e-hookah, flavored e-liquid is placed inside the bowl and heated electrically. Inhalation from the hose activates a pressure sensor—inside the e-hookah head—that turns on the heating coils, which in turn atomizes the flavored e-liquid. The aerosol travels down through the body of the device and into the water bowl before being carried through the hose into the user’s airway.

Large elastic artery stiffness, typically assessed by carotid-femoral pulse wave velocity (PWV) and central BP, is an independent risk factor for cardiovascular disease and all-cause mortality.^10^^,^^11^ In the community-based Framingham Heart Study, measures of arterial stiffness were associated with the risk of cardiovascular events.^12^ e-Cigarette vaping acutely increases arterial stiffness comparable with that of traditional cigarette smoking.^13^ We recently demonstrated that traditional hookah smoking acutely increases arterial stiffness.^14^ However, the comparable effect of e-hookah vaping has yet to be investigated.

In the present randomized crossover-design study, we investigated the acute differential effect of e-hookah vaping in comparison with traditional charcoal-heated hookah smoking on measures of conduit arterial stiffness, including carotid-femoral PWV, and augmentation index corrected for heart rate and central BP before and after a 30-min hookah smoking or vaping session. We assessed a panel of circulating biomarkers indicative of inflammation (high-sensitivity C-reactive protein [hsCRP], fibrinogen, and tumor necrosis factor α [TNFα]) and lipid peroxidation (paraoxonase-1 [PON-1] activity, arylesterase activity, and high-density lipoprotein [HDL] antioxidant capacity determined by an HDL oxidative index) and measured plasma nicotine and exhaled CO levels before and after the smoking or vaping sessions.

## Methods

### Study Design and Participants

The study population included healthy young habitual hookah smokers between 21 and 39 years of age who do not smoke cigarettes and met the following criteria: (1) no evidence of cardiopulmonary disease by history or physical examination; (2) BP < 140/90 mm Hg; (3) BMI of *>* 18.50 kg/m^2^ and < 30 kg/m^2^; (4) resting heart rate of < 100 beats/min; (5) take no prescription medication; (6) not pregnant (confirmed by urine test) or breastfeeding; (7) have smoked hookah at least 12 times in the past 12 months^15^; (8) have not smoked cigarettes in the past 12 months, smoked fewer than 100 cigarettes in their lifetimes, or both; (9) have not smoked marijuana in the past 12 months and showed negative results on a urine tetrahydrocannabinol screen; and (10) end-expiratory CO of < 10 parts per million before the study (evidence for no recent or current combusted tobacco exposure).

All participants agreed to fast for 8 h and abstain from exercise, antioxidants, caffeine, and alcohol for 48 h before the study. Participants were instructed not to smoke or vape e-hookah or any other electronic nicotine device, including e-cigarettes, and to avoid exposure to any secondhand smoke for 72 h before the study. The experimental protocol was approved by the University of California, Los Angeles, Medical Institutional Review Board 3 (Identifier: 18-001559), and informed written consent was obtained from all participants.

### e-Hookah Vaping and Combustible Hookah Smoking Sessions

Using an e-hookah (Starbuzz Wireless E-head, Starbuzz Tobacco, Inc.) placed on a traditional water pipe, participants were instructed to vape e-hookah fruit-flavored liquid mix containing a 50-50 blend of propylene glycol and vegetable glycerin and 6 mg/mL nicotine (Starbuzz Tobacco, Inc.). Because the e-hookah bowl has various power settings, based on participants’ preferences and reported use, the device power was set at 50 W.

For hookah smoking sessions, a traditional water pipe was used. Participants were instructed to smoke the most popular brand of maassel cited by hookah smokers and manufactured in the United States^16^ (5%-10% tobacco fermented with molasses, fruit, and glycerin; Starbuzz Tobacco, Inc.) heated with two charcoal briquettes (Coco Nara 100% Natural Coal, Coco Nara).

To mitigate the impact of carryover effects, sessions were separated by a 7-day washout period. Vaping and smoking topography were standardized in accordance with hookah smoking puffing parameters observed in natural settings.^17^^,^^18^ For the duration of the 30-min inhalation sessions, all participants were cued verbally to inhale a 3-s puff at 20-s intervals, with vapor remaining in the lungs for approximately 3 s of breath-holding after inhalation. Supervision was carried out to prevent superficial vaping or hyperventilation. Experimental sessions took place in a specifically designed smoking room within the University of California, Los Angeles, Clinical and Translational Research Center. All measurements were performed before and immediately after (*<* 10 min) the sessions.

### Arterial Stiffness Measurements

Carotid-femoral PWV was measured by simultaneous waveform capture using both a thigh-specific cuff and carotid artery applanation tonometry (SphygmoCor XCEL; AtCor Medical). Velocity (*d*_*sf*_ – *d*_*sc*_ (m) / time (s)) was calculated by measuring the time difference between the initial upstroke of the recorded waveforms at each site. The linear distance was measured manually from the suprasternal notch to the top of the thigh cuff at the center line of the leg, at the location of the femoral artery (*d*_*sf*_), and subtracting the distance from the suprasternal notch to the location of the carotid pulse (*d*_*sc*_). The transit time between the carotid and the femoral pulse waves was determined automatically by the SphygmoCor software.

The augmentation index and central BP were derived from the contour of the brachial BP waveform. The brachial-artery waveform, calibrated using oscillometric brachial artery BP, was analyzed by the validated brachial-to-aortic SphygmoCor transfer function to generate a central waveform and associated parameters.^19^ The augmentation index was calculated as the ratio of augmentation pressure (difference between the second and first systolic peaks of the aortic pressure waveform) and pulse pressure expressed as a percentage.

### Biomarkers of Inflammation and Oxidation

Blood samples were obtained from the antecubital vein, drawn into pre-iced heparinized vacutainers, and placed on ice. Three tubes were sent to the University of California, Los Angeles, Clinical Laboratory for inflammatory biomarker analyses, which were performed within 24 h after collection. One tube was centrifuged to separate plasma for antioxidant biomarker analyses, and samples were frozen at –80°C in a cryopreservative solution.^20^

Serum hsCRP levels were analyzed using the immunoturbidimetric method using the Siemens Vista Dimension analyzer, which provides a minimum detection level of < 0.2 mg/L. Quantitative determination of fibrinogen levels were determined by the clotting method (Clauss method). The within-assay coefficient of variation (CV) for fibrinogen is 2.8% to 3.7%, and the interassay CV is 1.2% to 3.0%. TNFα was analyzed using quantitative multiplex bead assay.

#### Arylesterase Activity

Activity was determined by the rate of hydrolysis of phenyl acetate to phenol, as described previously.^21^ Briefly, 4 μL plasma was incubated with 3.5 mM phenyl acetate in 9 mM Tris-HCl buffer (pH, 8.0) containing 0.9 mM CaCl_2_ at RT. The kinetics of phenol formation were determined by recording the absorbance at 270 nm every 15 s for 2 min. The activity was expressed as nanomoles of product formed per minute per milliliter of plasma.

#### PON-1 Activity

We determined the ability of PON-1, associated with HDL, to hydrolyze paraoxon substrate.^22^^,^^23^ The hydrolysis of paraoxon (diethyl-p-nitrophenyl phosphate) to p-nitrophenol by PON-1 was determined by incubating 5 μL of plasma with 1.0 mM paraoxon in 100 mM tris-HCl buffer (pH, 8.5).^24^ The kinetics of *p*-nitrophenol formation was determined by recording absorbance at 405 nm every 15 s for 4 min. The enzyme activity was expressed as micromoles of p-nitrophenol formed per minute for every 1 mL plasma and assayed in triplicates. The intra-assay CV for the assay was 2.60% and the interassay CV was 8.95%.

#### HDL Antioxidant Capacity

Capacity was determined as the ability of HDL to inhibit LDL-induced oxidation of dihydrodichlorofluorescein into the fluorescent dichlorofluorescein.^24^ Capacity was expressed as an HDL oxidative index, determined by the ratio of dichlorofluorescein fluorescence in the presence and absence of HDL and assayed in triplicates. An index of < 1.0 denotes protective antioxidant HDL, whereas an index of > 1.0 indicates pro-oxidant HDL.^20^ The within-assay CV was 6.89%. The interassay CV for four separate measurements over a period of 2 months was 7.30%.

### Biomarkers of Exposure

Plasma nicotine levels were assayed by gas chromatography with nitrogen-phosphorus detection, using 5-methylnicotine and 1-methyl-5-(2-pyridyl)-pyrrolidin-2-one (ortho-cotinine) as internal standards.^25^ Expired CO measurements were carried out using a CO meter (Micro Smokerlyzer; Bedfont Scientific Ltd.).

### Statistical Analysis

Paired Student *t* tests were used to compare continuous variables between sessions before and after exposure. Because of the crossover study design, we used a general linear model approach for repeated measures to examine differences between e-hookah and combustible hookah; the models included two within-subject factors (product type and time point relative to exposure session), and the sequence of type of product exposure was included as a between-group factor. The effect of primary interest was the interaction between the two within-subject factors, product type and time point relative to the exposure session. Effect sizes are for the interaction of product-by-time point relative to exposure in the general linear model repeated-measures analysis and translated from eta-square metric to d-metric.^26^ Statistical significance was set at .05 and analyses were conducted using SPSS Statistics version 24.0 software (IBM).

## Results

### Participant Characteristics

**Figure 2:**
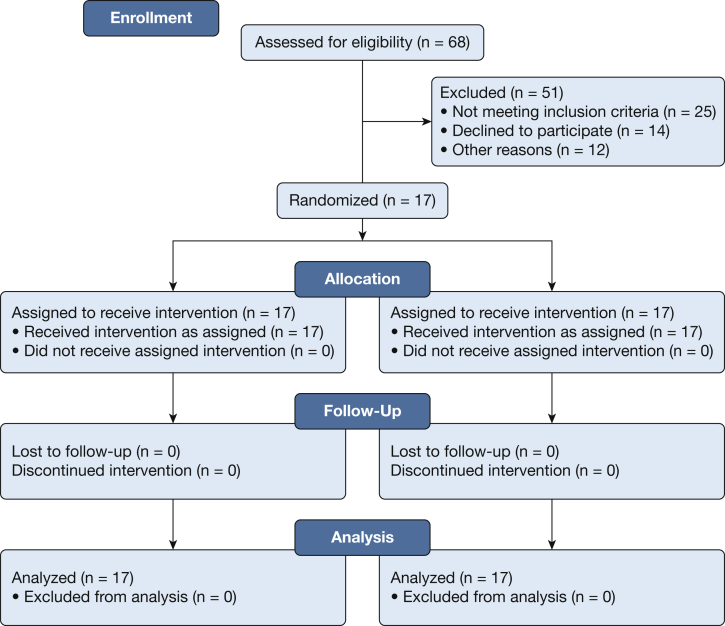
Consolidated Standards for Reporting Trials diagram showing the flow of included participants.

Sixty-eight potential participants responded to advertisement in local media, colleges, and universities, and 42 were screened for participation. Of these, 17 met study criteria. Twenty-five participants were excluded for the following reasons: positive tetrahydrocannabinol test results on screening (n = 7); history of cigarette or marijuana smoking, or both (n = 11); history of obesity or hypertension (n = 5); and exhaled CO of > 10 parts per million on screening (n = 2). The Consolidated Standards for Reporting Trials diagram is shown in Figure 2. Participant demographics are displayed in Table 1. Our sample mostly comprised college graduates who reported starting to smoke hookah flavored tobacco between 18 and 24 years of age, on average twice weekly for 5.6 ± 0.1 years.

**Table 1:** Participant Characteristics

Variable	Value
No.	17
Sex, female/male	5/12
Age, y	26.0 ± 1.1
BMI, kg/m^2^	24.8 ± 0.6
Race or ethnicity	
Non-Hispanic White	4
Non-Hispanic Black	4
Hispanic	1
Asian	5
Middle Eastern	3
Education	
High School	2
College	14
Graduate school	1
History of hookah smoking	
Smoking sessions, no. per wk	2 ± 1
Session duration, min	120.0 ± 10.3
Preferred hookah tobacco/liquid flavor	
Candy	4
Fruit	12
Alcohol	1
Menthol	0
Age of hookah smoking onset, y	
*<* 17	2
18-24	11
25-32	4

Data are presented as No. or mean ± SEM.

### Effect on BP and Arterial Stiffness

A total of 34 sessions were completed (17 e-hookah vaping and 17 hookah smoking sessions). Within 10 min after the sessions, both products acutely caused a significant increase in heart rate and brachial and central BP (*P* < .05) (Table 2). With e-hookah vaping sessions, although all participants achieved comparable acute increases in heart rate, BP, and measures of arterial stiffness, four participants reported experiencing throat irritation. Although central pulse pressure did not increase significantly after exposure to either product, e-hookah vaping led to a significant increase in pulse pressure, whereas combustible hookah smoking did not. e-Hookah vaping tended to lead to a higher increase in carotid-femoral PWV than combustible hookah smoking (mean ± SE: e-hookah, +0.74 ± 0.12 m/s; combustible hookah, +0.57 ± 0.14 m/s; *P* < .05 for both). Compared with baseline, only e-hookah vaping induced an acute increase in augmentation index (*P* = .004). Comparing baseline values of BP and arterial stiffness indexes between the two experimental sessions showed no significant differences.

**Table 2:** Hemodynamics, Peripheral and Central BP Changes, Arterial Stiffness Parameters, and Smoking Exposure Biomarkers Before and After the Smoking and Vaping Sessions

Variable	Electronic Hookah Vaping			Charcoal Hookah Smoking		
	Before	After	Change (After – Before)	Before	After	Change (After – Before)
Heart rate, beats/min	69 ± 2	78 ± 3^a^	+9 ± 3	68 ± 2	77 ± 3^a^	+9 ± 2
Brachial BP, mm Hg						
Systolic	113 ± 2	128 ± 3^a^	+15 ± 2	110 ± 2	119 ± 3^a^	+9 ± 2
Diastolic	69 ± 2	78 ± 3^a^	+9 ± 2	66 ± 2	73 ± 2^a^	+7 ± 1
Pulse pressure	44 ± 2	50 ± 2^a^	+6 ± 2	44 ± 2	46 ± 2	+2 ± 2
Mean arterial pressure	83 ± 2	94 ± 2^a^	+11 ± 2	80 ± 2	88 ± 2^a^	+8 ± 1
Central BP, mm Hg						
Systolic	101 ± 3	110 ± 3^a^	+9 ± 2	98 ± 2	106 ± 3^a^	+8 ± 2
Diastolic	69 ± 2	77 ± 3^a^	+8 ± 2	66 ± 2	74 ± 3^a^	+8 ± 1
Pulse pressure	31 ± 1	34 ± 2	+3 ± 1	31 ± 1	33 ± 1	+2 ± 1
Mean arterial pressure	80 ± 2	88 ± 3^a^	+8 ± 2	77 ± 2	87 ± 2^a^	+10 ± 2
Arterial stiffness parameters						
Augmentation index @ 75, %	7.97 ± 2.97	13.55 ± 3.24^a^	+5.58 ± 1.54	7.79 ± 2.54	10.66 ± 2.98	+2.87 ± 2.12
Carotid-femoral PWV, m/sec	8.20 ± 0.26	8.94 ± 0.33^a^	+0.74 ± 0.12	8.15 ± 0.20	8.71 ± 0.23^a^	+0.57 ± 0.1

Data are presented as mean ± SEM. PWV = pulse wave velocity.

a *P* < .05.

### Effect on Inflammatory and Antioxidant Biomarkers

**Figure 3:**
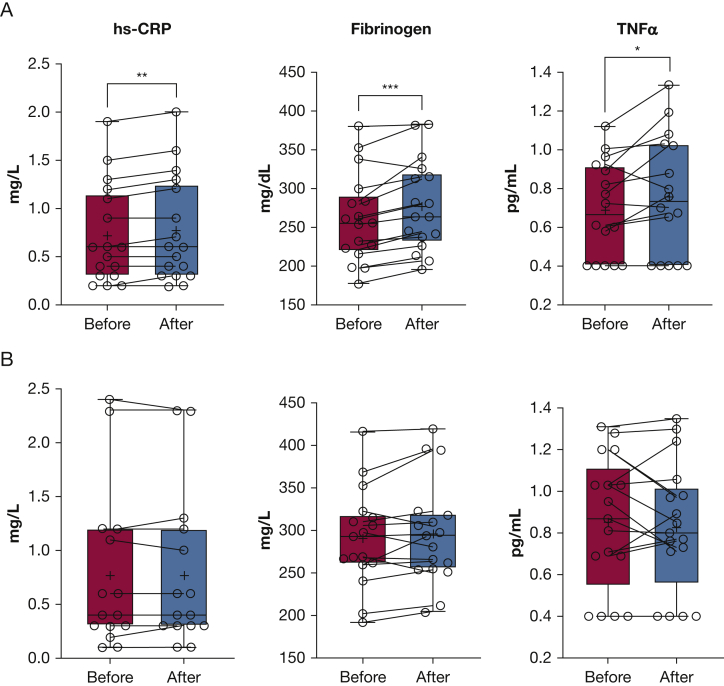
A, B, Representative boxplots showing levels of hsCRP, fibrinogen, and TNFα inflammatory biomarkers before and after exposure sessions: e-hookah vaping (A) and combustible hookah smoking (B). The solid horizontal line represents the median, the box represents the 25th to 75th percentiles, and the whiskers represent the 10th and 90th percentiles. ∗P = .025. ∗∗P = .005. ∗∗∗P = .001 (before vs after exposure). hsCRP = high sensitivity C-reactive protein; TNFα = tumor necrosis factor α.

**Figure 4:**
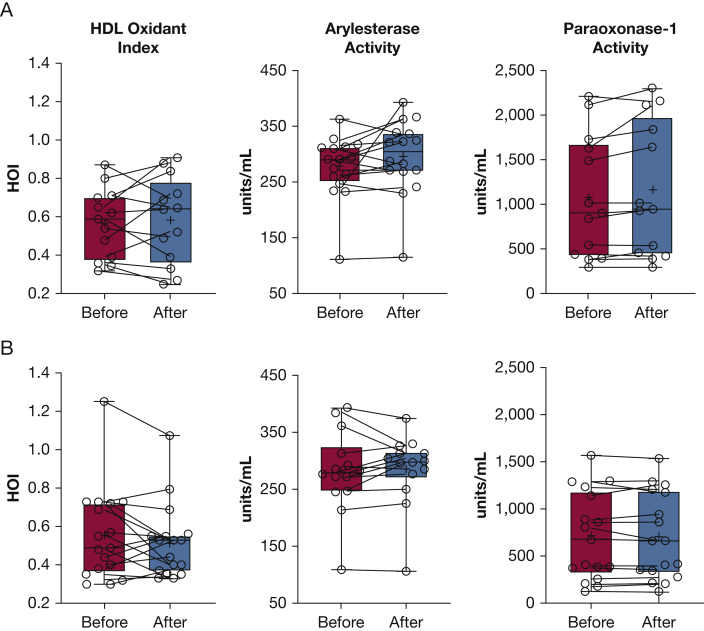
A, B, Representative boxplots showing of plasma levels of HOI, arylesterase activity, and paraoxonase-1 antioxidant biomarker activity before and after exposure sessions: e-hookah vaping (A) and combustible hookah smoking (B). The solid horizontal line represents the median, the box represents the 25th to 75th percentiles, and the whiskers represent the 10th and 90th percentiles. HOI = high-density lipoprotein oxidative index.

In comparison with baseline, the plasma proinflammatory biomarkers hsCRP, fibrinogen, and TNFα significantly increased acutely after e-hookah vaping (from 0.72 ± 0.12 mg/L to 0.76 ± 0.13 mg/L; from 261.41 ± 13.84 mg/dL to 277.47 ± 14.09 mg/dL; from 0.69 ± 0.06 pg/mL to 0.76 ± 0.08 pg/mL, respectively; all *P* < .05), but not after combustible hookah smoking (from 0.77 ± 0.19 mg/L to 0.77 ± 0.19 mg/L; from 290.82 ± 13.76 mg/dL to 296.59 ± 14.80 mg/dL; from 0.85 ± 0.08 pg/mL to 0.82 ± 0.08 pg/mL, respectively; all *P* values were not significant) (Fig 3A, 3B). Although changes from before to after exposure were significantly different between the two types of products for TNFα (*P* = .005), but not for hsCRP or fibrinogen (both *P* values were not significant), effect sizes were large for all three measures (*d* = 1.70, *d* = 1.09, and *d* = 1.05, respectively). Antioxidant biomarkers did not change after using either products (Fig 4A, 4B). Comparing baseline values of both inflammatory and antioxidant biomarkers between the two experimental sessions showed no significant differences (.10 *< P* < .95).

### Effect on Smoking Exposure Biomarkers

**Figure 5:**
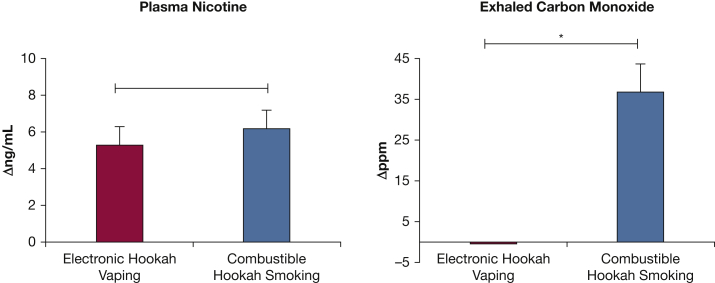
A, B, Bar graphs showing exposure biomarkers in response to acute e-hookah vaping and combustible hookah smoking for changes in plasma nicotine (A) and exhaled carbon monoxide (B) levels. ∗P < .005.

Exhaled CO levels were significantly higher after combustible hookah smoking than after e-hookah vaping (*P* < .001) (Fig 5). No difference was found between plasma nicotine concentrations after using either product (*P* = .478).

## Discussion

The increase in flavored hookah tobacco smoking among youth and young adults is global.^1^^,^^2^ Coinciding with the remarkable increase in electronic nicotine delivery systems (ENDSs), in 2014, the hookah tobacco companies introduced a putatively safe electronic alternative to traditional charcoal-heated hookah smoking, accompanied by unsubstantiated marketing claims that the presence of water “filters out toxins.”^27^^,^^28^ In a randomized crossover study among a sample of young healthy adult chronic hookah smokers, we found that e-hookah vaping tended to evoke a higher acute increase in carotid-femoral PWV than combustible hookah smoking. With e-hookah vaping, increases in carotid-femoral PWV were accompanied by acute elevations in the proinflammatory markers hsCRP, fibrinogen, and TNFα, whereas no changes were observed after hookah smoking. These data suggest that although advertised to be “safe,” e-hookah vaping exerts injurious effects on the vasculature which are, at least in part, mediated by inflammation.

The data herein extend prior studies implicating the unfavorable vascular and respiratory effects of ENDS among young, healthy adults. To date, virtually all translational research studies of ENDS have focused on e-cigarettes; consequently, almost nothing is known about e-hookahs. e-Cigarette vaping increases carotid-femoral PWV^13^ and, similar to cigarette smoking, negatively affects lung function^29^ and increases measures of airway resistance, including impedance, respiratory resistance, and peripheral airway resistance.^30^^,^^31^ When extrapolating from e-cigarette data to other ENDS such as e-hookahs, one must consider key differences between products, including design, battery size, voltage options, nicotine concentration, and flavoring constituents.^3^ Unlike e-cigarettes, with e-hookahs, the aerosol first passes through the traditionally designed water bowl, cooling and potentially altering the aerosol, before inhalation. Although the increase in hookah use is fueled in part by the unsubstantiated belief that smoke is filtered when passes through water, this concept is incorrect,^32^^,^^33^ because bubbles of smoke pass quickly through the water with little dissolution of smoke constituents, and reports indicate only a small effect of water filtration on nicotine, with less than 5% being trapped in the water.^33^^,^^34^

Because of the long hose and beyond the higher humidity of the smoke, hookah smokers take longer and deeper puffs, resulting in thicker smoke, compared with the shorter and lighter puffs associated with cigarette use.^35^ e-Hookah are designed to withstand high-voltage settings (up to 50 W),^4^ which has implications for formation of higher amounts of reactive free radicals and carbonyls, compared with devices with low voltage.^36^^,^^37^

The higher increases in PWV with e-hookah vaping, compared with hookah smoking, accompanied by acute elevations in proinflammatory markers presumably could be explained by the changes in CO and nicotine levels. CO, a principal byproduct of heme catabolism by heme oxygenases and a key molecule emitted from charcoal combustion with hookah smoking,^2^ has a protective role in vascular injury^38^ by exerting potent antiinflammatory effects and inhibiting production of TNFα and C-reactive protein expression.^39^, ^40^, ^41^ However, in the absence of other constituents, nicotine exposure induces proinflammatory C-reactive protein and TNFα expression^42^^,^^43^ and impairs arterial compliance by increasing carotid-femoral PWV, even after adjustment for changes in mean arterial pressure and heart rate.^44^ In our study, plasma nicotine concentrations were comparable, but the CO boost was 38-fold greater after combustible hookah smoking than after e-hookah vaping. With hookah smoking, it is possible that the large CO boost overpowered the nicotine-induced proinflammatory vascular effects, whereas with the absence of CO with e-hookah vaping, nicotine predominates the vascular effects. In support of this hypothesis is the finding that short-term exposure to charcoal-heated hookah smoke (30 min/day for 2 consecutive weeks) did not affect the plasma concentrations of C-reactive protein and TNFα.^45^ However, although our findings support the idea that CO is an unavoidable key molecule that should be considered for further cardiopulmonary studies comparing vaping (virtually no CO exposure) vs combustible technologies, it should be noted that nicotine also has been demonstrated to inhibit the production of proinflammatory mediators by suppressing phosphorylation of I-κBα and the transcriptional activity of nuclear factor kappa-B (NF-κB).^46^^,^^47^ Therefore, it is plausible that other nonnicotine components present in ENDS aerosols, including oxidizing chemicals, aldehydes (especially acrolein), and particulates,^48^ could contribute to the observed vascular proinflammatory changes.

In our study, we found that neither e-hookah vaping nor hookah smoking induced a detectable acute effect on oxidative stress burden. Carnevale et al^49^ demonstrated that e-cigarette vaping acutely raised 8-iso-prostaglandin F2α. We measured PON-1 because of its strong correlation with atherosclerosis and cardiovascular disease risk, with evidence showing its reduced activity in combustible cigarette smokers,^50^ and after subacute exposure to air pollution.^21^ It is plausible that potential homeostatic compensatory changes induced by chronic hookah use could have blunted acute oxidative responses, detectable by the biomarkers used in our present study.

## Interpretation

Our study has several limitations. It remains unclear whether the observed vascular effects are related to the effects of nicotine, oxidants, particulates, or a combination thereof, a question that is outside this study’s intended scope. Future studies should focus on elucidating the relative contribution of major constituents of vaping (ie, nicotine vs propylene glycol, glycerol, and flavorings) on mediating the observed vascular toxicity. Our study participants are overtly healthy young adults free of cardiovascular disease; thus, our findings cannot be extrapolated to others with underlying clinical conditions. Because our participants are chronic hookah smokers, such that they might have blunted effects of hookah smoking, future studies among tobacco-naïve participants or occasional hookah smokers might yield different results. Although it is presumed that the observed acute effects may add a burden to vascular health over time, the long-term effects, including later time points after exposure to assess for nonacute effects, of e-hookah vaping remain an open question. The absence of a nonsmoking or nonvaping, or both, control group did not allow for determining baseline vascular differences comparing the experimental groups. With our experimental studies, participant blinding was impossible during data collection, but offline data analyses were performed by blinded evaluators. Because we studied hookah products manufactured in the United States that are used among participants representing more of a US population, our findings cannot be extrapolated to Middle Eastern hookah products and populations. Finally, the relatively small number of hookah participants who were studied along with the strict criteria of study eligibility limit extrapolation of the study findings to the larger general population of hookah smokers overall.

Despite these limitations, our findings call into question the unsubstantiated social media and marketing claims that the presence of water “filters out toxins” with the use of a water pipe.^27^^,^^28^ Our study provides no physiological evidence that e-hookah vaping is a safe tobacco alternative; rather, in fact, it acutely impairs arterial elasticity, evoking systemic inflammation. Our findings may provide a scientific basis needed to inform tobacco regulatory science for the development of national policy regulation specific to hookah.

**Take-home Points**

**Study Question:** What is the differential acute effect of e-hookah vaping vs combustible hookah smoking on oxidation, inflammation, and arterial stiffness?

**Results:** Compared with combustible hookah smoking, e-hookah vaping tended to lead to a larger increase in large artery stiffening, accompanied by elevation of the proinflammatory biomarkers high-sensitivity C-reactive protein, fibrinogen, and tumor necrosis factor α.

**Interpretation:** Although advertised to be “safe,” flavored e-hookah vaping exerts injurious effects on the vasculature that are, at least in part, mediated by inflammation.
